# Protective effects of pyrroloquinoline quinone in brain folate deficiency

**DOI:** 10.1186/s12987-023-00488-3

**Published:** 2023-11-20

**Authors:** Vishal Sangha, Sara Aboulhassane, Qing Rui Qu, Reina Bendayan

**Affiliations:** Leslie Dan Faculty of Pharmacy, University of Toronto, Toronto, Canada

**Keywords:** Pyrroloquinoline quinone, Reduced folate carrier, Proton-coupled folate transporter, Neuroinflammation, Oxidative stress, Mitochondrial function, Cerebral folate deficiency

## Abstract

**Background:**

Folates (Vitamin B9) are critical for normal neurodevelopment and function, with transport mediated by three major pathways: folate receptor alpha (FRα), proton-coupled folate transporter (PCFT), and reduced folate carrier (RFC). Cerebral folate uptake primarily occurs at the blood-cerebrospinal fluid barrier (BCSFB) through concerted actions of FRα and PCFT, with impaired folate transport resulting in the neurological disorder cerebral folate deficiency (CFD). Increasing evidence suggests that disorders associated with CFD also present with neuroinflammation, oxidative stress, and mitochondrial dysfunction, however the role of brain folate deficiency in inducing these abnormalities is not well-understood. Our laboratory has identified the upregulation of RFC by nuclear respiratory factor 1 (NRF-1) at the blood–brain barrier (BBB) once indirectly activated by the natural compound pyrroloquinoline quinone (PQQ). PQQ is also of interest due to its anti-inflammatory, antioxidant, and mitochondrial biogenesis effects. In this study, we examined the effects of folate deficiency and PQQ treatment on inflammatory and oxidative stress responses, and changes in mitochondrial function.

**Methods:**

Primary cultures of mouse mixed glial cells exposed to folate-deficient (FD) conditions and treated with PQQ were analyzed for changes in gene expression of the folate transporters, inflammatory markers, oxidative stress markers, and mitochondrial DNA (mtDNA) content through qPCR analysis. Changes in cellular reactive oxygen species (ROS) levels were analyzed in vitro through a DCFDA assay. Wildtype (C57BL6/N) mice exposed to FD (0 mg/kg folate), or control (2 mg/kg folate) diets underwent a 10-day (20 mg/kg/day) PQQ treatment regimen and brain tissues were collected and analyzed.

**Results:**

Folate deficiency resulted in increased expression of inflammatory and oxidative stress markers in vitro and in vivo, with increased cellular ROS levels observed in mixed glial cells as well as a reduction of mitochondrial DNA (mtDNA) content observed in FD mixed glial cells. PQQ treatment was able to reverse these changes, while increasing RFC expression through activation of the PGC-1α/NRF-1 signaling pathway.

**Conclusion:**

These results demonstrate the effects of brain folate deficiency, which may contribute to the neurological deficits commonly seen in disorders of CFD. PQQ may represent a novel treatment strategy for disorders associated with CFD, as it can increase folate uptake, while in parallel reversing many abnormalities that arise with brain folate deficiency.

**Supplementary Information:**

The online version contains supplementary material available at 10.1186/s12987-023-00488-3.

## Background

Folates (or vitamin B9) serve as one-carbon donors in various biosynthetic and homeostatic processes critical for cellular growth and development [1]. In mammals, the uptake of folate derivatives is mediated by three distinct transport mechanisms that vary in affinity and optimal activity conditions [2–4]. The folate receptors (gene: *FOLR1-2*/*Folr1-2*) are high affinity glycosylphosphatidylinositol-linked membrane proteins, which function through receptor-mediated endocytosis at pH 7.4 [3–6]. The reduced folate carrier (RFC) (gene: *SLC19A1*/*Slc19a1*) is an antiporter with intracellular organic phosphates and operates at physiological pH [2, 4, 7]. Lastly, the proton-coupled folate transporter (PCFT) (gene: *SLC46A1*/*Slc46a1*) functions as a proton cotransporter, with optimal activity observed at pH 4.5–5.5 [2]. Due to its optimal activity conditions, PCFT plays a major role in the intestinal absorption of folates [2].

Within the central nervous system (CNS), folate requirements exceed those typically seen in peripheral tissues, thus requiring an effective folate transport system to be in place to meet the metabolic demands of the CNS [8]. Cerebral folate uptake primarily occurs at the blood-cerebrospinal fluid barrier (BCSFB), through coordinated actions of folate receptor alpha (FRα) and PCFT [9, 10]. Inadequate folate transport at this interface can result in cerebral folate deficiency (CFD) syndromes, which are characterized by suboptimal CSF folate concentrations, despite normal systemic levels [9, 11]. Impaired FRα function can arise due to mutations in the *FOLR1* gene encoding FRα, or through the presence of autoantibodies against FRα (FRAAs), both resulting in impaired folate transport across the choroid plexus epithelium [12–14]. More recently, the presence of FRAAs (classified as secondary CFD) has been identified in many other neurological disorders such as autism spectrum disorder (ASD), Rett syndrome, Alper’s syndrome, and Kearns-Sayre syndrome (KSS) [15–18].

The Bendayan lab has uncovered the blood–brain barrier (BBB) as an effective alternate route of folate delivery in the context of CFD, mediated through RFC [19–22]. Notably, our group has demonstrated that activation of the nuclear receptor Vitamin D receptor (VDR) by its specific ligand 1,25-dihydroxyvitamin D3 (or calcitriol) resulted in significant increases in RFC expression and activity at the BBB which could effectively restore brain folate concentrations in mice lacking Frα (a model reflective of CFD) [19, 20]. More recently, we identified nuclear respiratory factor 1 (NRF-1) as an additional transcription factor that may modulate RFC expression and function [21, 22]. NRF-1 has been previously recognized for its role in regulating key processes in mitochondrial biogenesis and respiratory function and can be indirectly activated by the naturally derived enzyme cofactor pyrroloquinoline quinone (PQQ) through peroxisome proliferator-activated receptor-γ coactivator-1α (PGC-1α) signaling, of which NRF-1 is a downstream target [23–25]. We’ve shown that NRF-1 activation by PQQ results in enhanced RFC expression in vitro, in human brain microvessel endothelial cells (hCMEC/d3 cells), and in vivo in isolated mouse brain capillaries, representative of the mouse BBB [21, 22]. Beyond the choroid plexus epithelium and BBB, our recent studies have identified the arachnoid barrier (AB) as an additional blood-CSF barrier that may facilitate folate uptake into the CNS, with evidence of RFC and PCFT localization and expression in AB epithelial cells [26].

PQQ has been a compound of interest as several studies have demonstrated its anti-inflammatory and antioxidant effects, in addition to its role in improving mitochondrial function both in vitro and in vivo [27–32]. Of particular interest is the role of PQQ in the CNS, as it has shown to elicit neuroprotective effects in several models of brain injury and disease [29, 30, 33].

Since adequate folate concentrations are required for several key biosynthetic processes, folate deficiency has also been implicated in the enhancement of inflammatory and oxidative stress responses [34–37]. Due to the role of folates in DNA synthesis and repair, folate deficiency has also been associated with mitochondrial dysfunction through increased mitochondrial DNA (mtDNA) deletions and decreased overall mtDNA content, as well as impaired activity of mtDNA-encoded enzymes which may contribute to impaired cellular respiration [36, 38].

Emerging evidence suggests that mitochondrial dysfunction, oxidative stress, and inflammation characterize many neurological disorders that have also been associated with CFD such as ASD, Rett syndrome, Alper’s syndrome and KSS [39, 40]. In disorders associated with FRAAs and low CSF folate such as ASD and Rett syndrome, mitochondrial dysfunction has been observed through the downregulation of mitochondrial enzymes and increased mtDNA deletions, along with increases in oxidative stress markers [18, 41, 42]. Lastly, inflammation has been documented in these disorders, through elevations of CSF inflammatory markers and microglial activation [43, 44]. While these various abnormalities have been increasingly recognized in disorders of CFD, the contribution of brain folate deficiency in inducing these changes is not well understood. We have previously demonstrated the role of PQQ in inducing RFC functional expression at the BBB, mediated through PGC-1α/NRF-1 signaling [21, 22]. We aim to further our understanding of the role of PQQ in the regulation of the folate transport systems in the context of CFD, as well as its neuroprotective effects. The objective of the present study is to assess the role of folate deficiency in inducing changes in inflammation, oxidative stress, and mitochondrial dysfunction, and PQQ’s role in reversing these effects while in parallel upregulating folate transporter expression. Further understanding the contribution of folate deficiency in inducing these pathophysiological changes will aid in the development of novel treatment strategies for disorders associated with CFD.

## Materials and methods

### Materials

All cell culture reagents used for in vitro experiments were of the highest quality and purchased from Invitrogen (Carlsbad, CA, USA) unless indicated otherwise. Pyrroloquinoline quinone or PQQ was obtained from Cayman Chemical (Ann Arbor, MI, USA). Real time quantitative polymerase chain reaction (qPCR) reagents were purchased from Applied Biosystems (Foster City, CA, USA). Primers for qPCR analysis were purchased from Life technologies and were validated for use with TaqMan qPCR chemistry (Carlsbad, CA, USA). The 2′,7′-dichlorofluorescein (DCFH) cellular ROS assay kit (ab113851) was purchased from Abcam (Cambridge, UK). Custom DNA oligonucleotides for mtDNA assays were purchased from Thermo Fisher Scientific (Waltham, MA, USA).

### Cell culture

For our in vitro experiments, we utilized primary cultures of mouse mixed glial cells, which have been routinely used in our laboratory to examine changes in inflammation and oxidative stress in response to various neurotoxic compounds [45, 46]. As astrocytes and microglia play a critical role in maintaining CNS homeostasis and immune regulation, they were deemed an appropriate in vitro model for our studies [47, 48]. Primary cultures of mouse mixed astrocytes and microglia (or mixed glial cells) were prepared in our laboratory as previously described [46]*.* In brief, cerebral cortices were dissected from 1 to 2-day old C57BL6/N mice pups, and brains were further processed to generate a mixed glial suspension. Cell suspensions from four brains were plated onto 75 cm^2^ poly-D-lysine coated tissue flasks and incubated in a complete media consisting of DMEM (Wisent Inc, Montreal, QC, Canada) supplemented with 10% heat inactivated fetal bovine serum (FBS), and 1 × penicillin–streptomycin at 37 ℃ in 5% CO_2_/95% air. For folate-deficient (FD) studies, cells were grown in complete medium lacking folic acid (Wisent Inc, Montreal, QC, Canada). To confirm folate concentrations in control and FD media, a microbiological assay was performed using *Lactobacillus rhamnosus* (control media: 2.82 µg/ml, FD media: 0.08 ng/ml) [49]. Upon reaching confluence, cells were collected and further processed for downstream analysis. Mixed glial cells were characterized by analyzing gene expression of the standard astrocyte and microglial markers GFAP and IBA1, respectively.

### Mouse model of folate deficiency

All procedures were performed in accordance with the Canadian Council on Animal Care guidelines and approved by the University of Toronto Animal Care Committee. To examine the in vivo effects of folate deficiency, an l-amino acid defined diet containing 0 mg/kg folic acid (supplemented with 1% succinyl sulfathiazole) was formulated, with an L-amino acid defined diet containing 2 mg/kg folic acid designated as the control diet (Envigo, Indianapolis, IN, USA). 24 Male wildtype C57BL6/N mice (aged 3–4 weeks) were randomly assigned to the control or FD diet (12 animals per diet group) for a 5-week period. All animals were allowed *ad-libitum* access to food and water. We confirmed cerebral folate deficiency in mice assigned to FD diets by measuring folate levels in mouse brains applying the microbiological assay [49]. This FD diet is like the one used in the study by Chou et al. where rats placed on diets lacking folic acid for 4 weeks showed significant reductions in brain folate levels, increased oxidative stress, and changes in mitochondrial function [36]. Following the end of the 5-week period, mice underwent a 10-day PQQ (or saline control) treatment period (6 animals per treatment group), with relevant control or FD diets maintained throughout this treatment period. This 10-day treatment period led to loss of an experimental animal in the PQQ control and saline FD group, respectively (saline control: N = 6, PQQ control: N = 5, saline FD: N = 5, PQQ FD: N = 6). 24-h following the last injection, animals were anaesthetised through isoflurane inhalation and exsanguination was performed by cardiac puncture. Animals were later decapitated and whole brains were collected for gene expression analysis and measurement of brain folate levels.

### PQQ treatments

For in vitro experiments, confluent mixed glial cells on 25 cm^2^ flasks were treated with PQQ (5 µM) or dimethyl sulfoxide (DMSO) vehicle (~ 0.08% DMSO in control or FD media) for 24 or 48 h at 37 ℃. Following the desired time interval, cells were collected and processed for analysis. Due to the low yield of cells typically observed from these culture preparations, experiments were conducted with a single concentration of PQQ (5 µM). Doses from 1 to 10 µM have been previously used in several in vitro studies demonstrating the anti-inflammatory and antioxidant effects of PQQ, as well as its effects on increasing mitochondrial function [25, 30, 32]. In addition, in our previous NRF-1 studies, hCMEC/D3 cells treated with 5 µM PQQ resulted in significant increases in RFC and PGC-1α expression [21, 22]. To confirm that there was no toxicity observed at this selected concentration, a MTT assay was performed on control and FD cells (Additional file 1: Fig. S1). For in vivo experiments, male C57BL6/N mice (8–12 weeks old) were subjected to daily intraperitoneal (i.p.) injections with PQQ (20 mg/kg dissolved in saline) or a saline vehicle for 10 consecutive days. Mice were anesthetized 24 h following the final injection through isoflurane inhalation and were later decapitated for brain tissue collection. This regimen is similar to the one employed by Cheng et al. where a 20 mg/kg/day PQQ treatment for 21 consecutive days exhibited neuroprotective effects in a rotenone-induced Parkinson’s disease mouse model, while upregulating the expression of NRF-1 target genes [50].

### Gene expression analysis

mRNA expression of the various genes of interest was assessed in cells and tissue using qPCR analysis. Total RNA was isolated from primary cultures of mouse mixed glial cells and mouse brain tissue using TRIzol and treated with DNase I to remove any contaminating genomic DNA. RNA concentration (absorbance at 260 nm) and purity (absorbance ratio 260/280) was assessed using NanoDrop One Spectrophotometer (Thermo Fisher Scientific, Waltham, MA, USA). An absorbance 260/280 ratio of ~ 2 was deemed acceptable for RNA samples. Following isolation, RNA (2 μg) was reverse transcribed to cDNA using a high-capacity reverse transcription cDNA kit according to manufacturer’s instructions. Specific mouse primers for: *Slc19a1* (RFC), *Slc46a1* (PCFT), *Folr1* (FRα), *Gfap* (GFAP), *Aif1* (Iba1); *Nrf1* (NRF-1), *PPargc1a* (PGC-1α), *Tfam* (Tfam), *Tfb1m* (TFB1M), *Tfb2m* (TFB2M), *Nos2* (iNOS), *Il6* (IL-6), *Il1b* (IL-1β), *Ccl3* (CCL3), *Cxcl10* (CXCL10) were obtained from Life Technologies for use with TaqMan qPCR chemistry. All gene expression assays were performed in triplicates using the housekeeping gene cyclophilin B as the internal control. For each gene of interest, relative mRNA expression was determined by calculating the difference in CT values (ΔCT) between the target gene and cyclophilin B.

### Reactive oxygen species measurement

The presence of ROS in vitro, a marker of oxidative stress, was detected using the 2′,7′-dichlorofluorescein (DCFH) cellular ROS assay kit (ab113851) according to manufacturer’s protocol (Abcam, Cambridge, USA). Briefly, confluent mouse mixed glial cells grown on 25 cm^2^ flasks in FD media and complete media (as a control) were seeded onto a 96-well plate (25,00 cells per well) in conditions of PQQ (5 µM) or DMSO vehicle (~ 0.08% DMSO in control or FD media) treatment for 24 h. Cells were then incubated with dichlorodihydrofluorescein diacetate (DCFDA), and fluorescence was detected on a microplate reader at excitation and emission wavelengths of 485 nm and 535 nm, respectively. Readings were subtracted from a negative control containing no cells and were normalized as a percentage relative to the DMSO control.

### mtDNA content measurement

Changes in mtDNA content was assessed by analyzing the mtDNA/nuclear DNA (nDNA) ratio as described in Quiros et al. [51]. Briefly, confluent mouse mixed glial cells grown on 25 cm^2^ flasks in FD media or complete media (as a control) in conditions of PQQ treatment or vehicle treatment were collected and genomic DNA was purified using the Genomic DNA purification kit according to manufacturer’s protocol. (Thermofisher Scientific, Waltham, MA, USA). Primers for the mtDNA-encoded NADH-ubiquinone oxidoreductase chain 1 (ND1), 16S ribosomal RNA (16S) and nuclear DNA-encoded hexokinase 2 (HK2) were developed as described in Quiros et al. and then coamplified by qPCR. The ratio of mtDNA to nuclear DNA was determined by the following equation: ΔC_t_ = C_tmtND1_—C_tHK2_ [51].

### Statistical analysis

All statistical analysis was performed using Prism 9 software (GraphPad Software Inc., San Diego, CA, United States). For all in vitro work, experiments were repeated at least three times using separate primary cultures of mixed glial preparations. For in vivo experiments, tissue samples were collected from 5 to 6 animals per treatment group. Results are presented as mean ± SEM. Multiple group comparisons were examined using one-way analysis of variance (ANOVA) with Bonferroni’s post hoc test, with P < 0.05 being considered statistically significant.

## Results

### Effects of folate-deficiency and PQQ treatment on transporter expression in primary cultures of mouse mixed glial cells

**Fig. 1:**
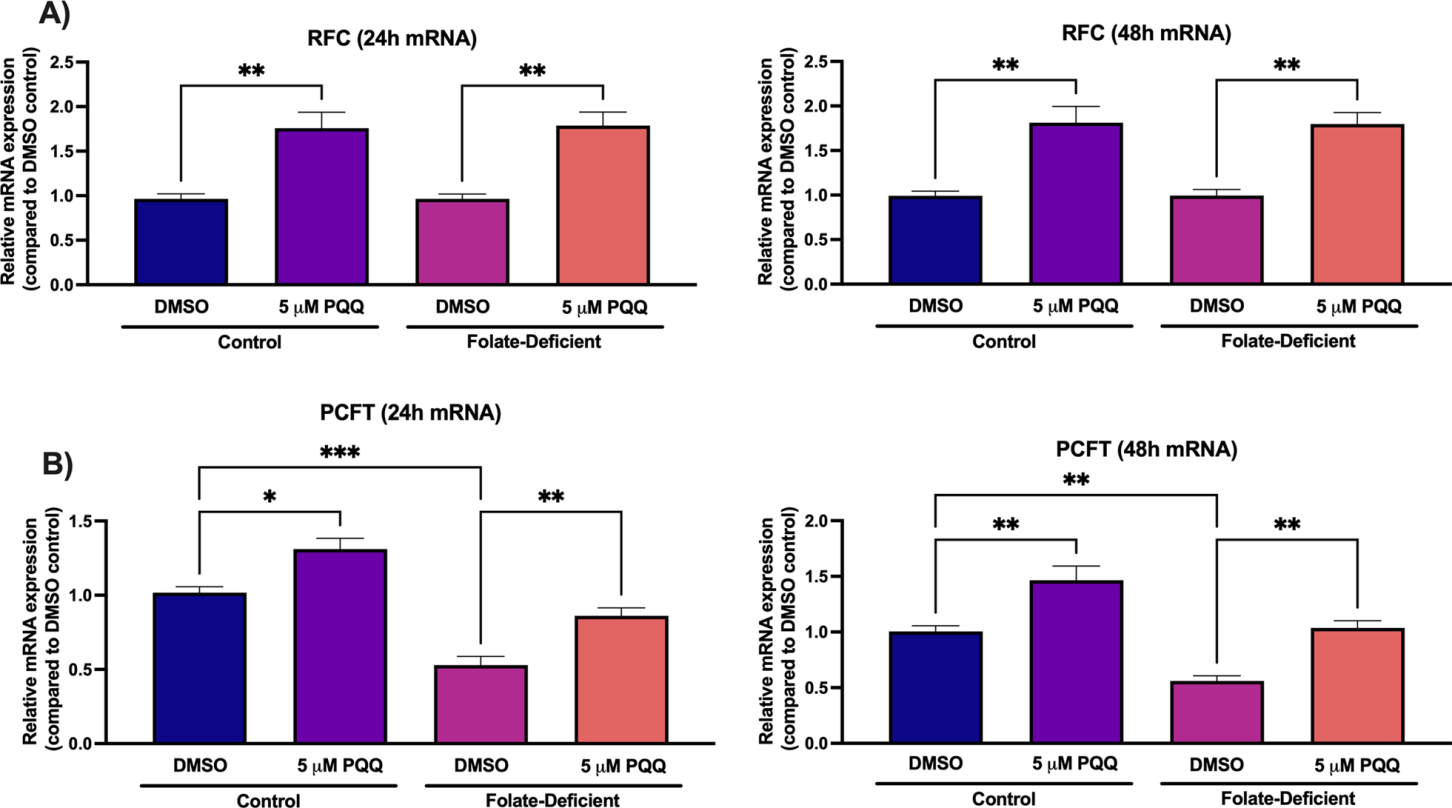
Effect of PQQ treatment on folate transporters RFC (*Slc19a1*) and PCFT (*Slc46a1*) gene expression in primary cultures of mouse mixed glial cells. **A** RFC gene expression was significantly increased in both control and FD cells following 24- and 48- hours PQQ treatment (5 µm).** B** PCFT gene expression was significantly lower in FD cells compared to control cells. PCFT gene expression was significantly increased in both control and FD cells following 24- and 48-h PQQ treatment (5 µm). Results are presented as mean relative mRNA expression compared to the DMSO vehicle control ± SEM normalized to the housekeeping gene cyclophilin B from N = 5 independent primary cultures of mouse mixed glial preparations. One-way ANOVA with Bonferroni’s post-hoc test. Asterisks represent significant differences (*P < 0.05, **P < 0.01, ***P < 0.001)

We have previously demonstrated the regulation of RFC by PGC-1α/NRF-1 signaling once activated by PQQ at the BBB [21, 22]. The present study further examined the regulation of the folate transport systems by PQQ in the context of brain folate deficiency in brain parenchymal glial cells. Through qPCR analysis, changes in expression of the folate transport systems were assessed. In cells exposed to FD conditions, gene expression levels of PCFT (but not RFC) were significantly reduced (~ 50%) compared to cells grown in control media (Fig. 1). Additionally, FRα gene expression in both control and FD mixed glial cells was minimal (data not shown), which is in line with our previous studies [26]. Cells treated with PQQ (24 and 48 h) displayed significant increases in both RFC and PCFT gene expression. Following 24 and 48-h treatments, RFC gene expression was increased by ~ 75% in both control and FD cells (Fig. 1A). Following the 24-h PQQ treatment, PCFT expression was increased by ~ 30% in control and FD cells (Fig. 1B). Following the 48-h PQQ treatment, PCFT expression was increased by ~ 50% in control and FD cells (Fig. 1B).

### Effects of folate deficiency and PQQ treatment on inflammation and oxidative stress in mixed glial cells

**Fig. 2:**
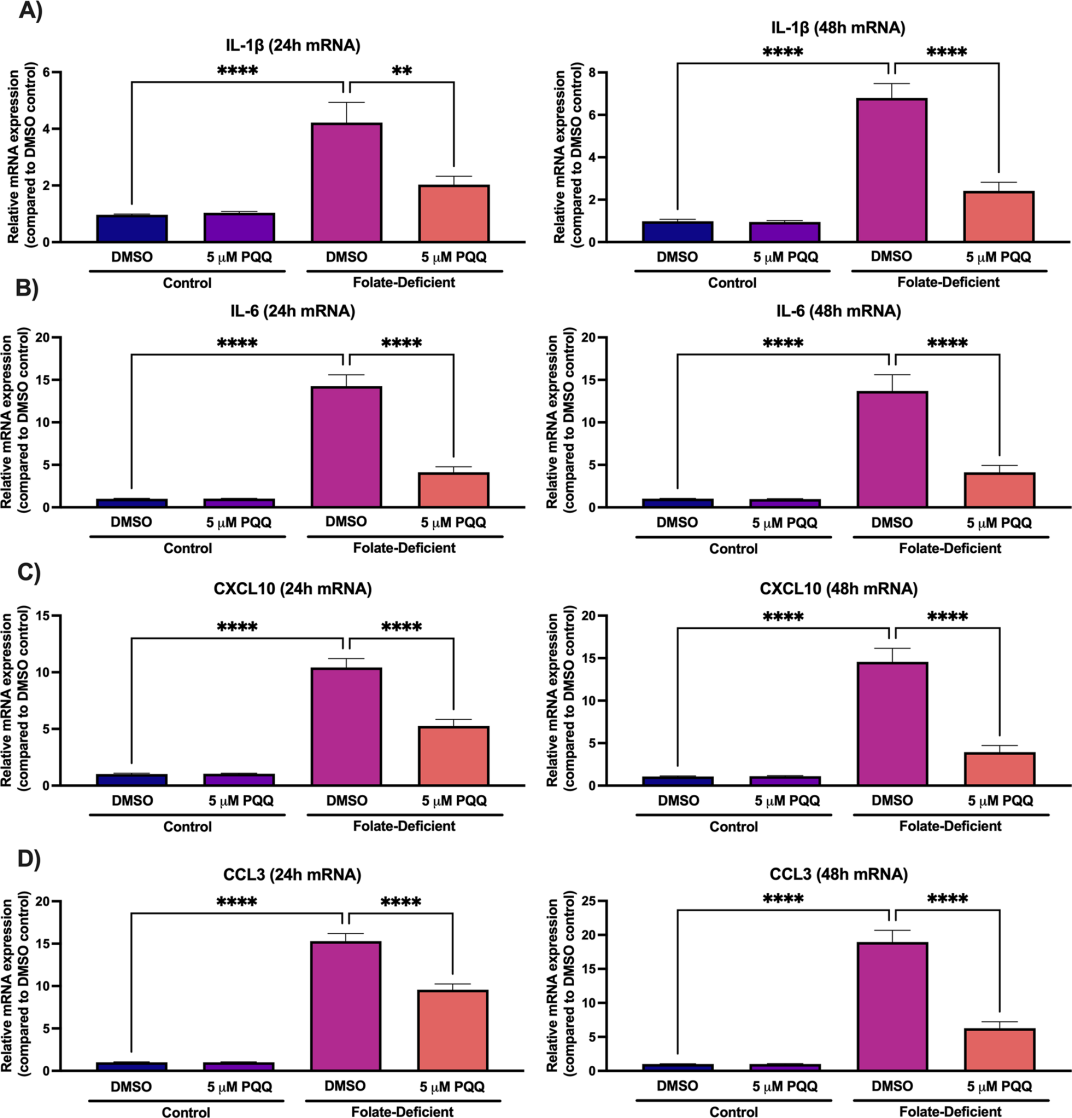
Effect of folate deficiency on inflammatory marker gene expression in primary cultures of mouse mixed glial cells. **A**-**D** IL-1β, IL-6, CXCL10, and CCL3 gene expression was significantly increased in FD cells compared to control cells, with a significant reduction in gene expression observed in FD cells following 24- and 48-h PQQ treatment (5 µm) compared to vehicle. Results are presented as mean relative mRNA expression compared to the DMSO vehicle control ± SEM normalized to the housekeeping gene cyclophilin B from N = 5 independent mixed glial preparations. One-way ANOVA with Bonferroni’s post-hoc test. Asterisks represent significant differences (*P < 0.05, **P < 0.01, ***P < 0.001, ****P < 0.0001)

**Fig. 3:**
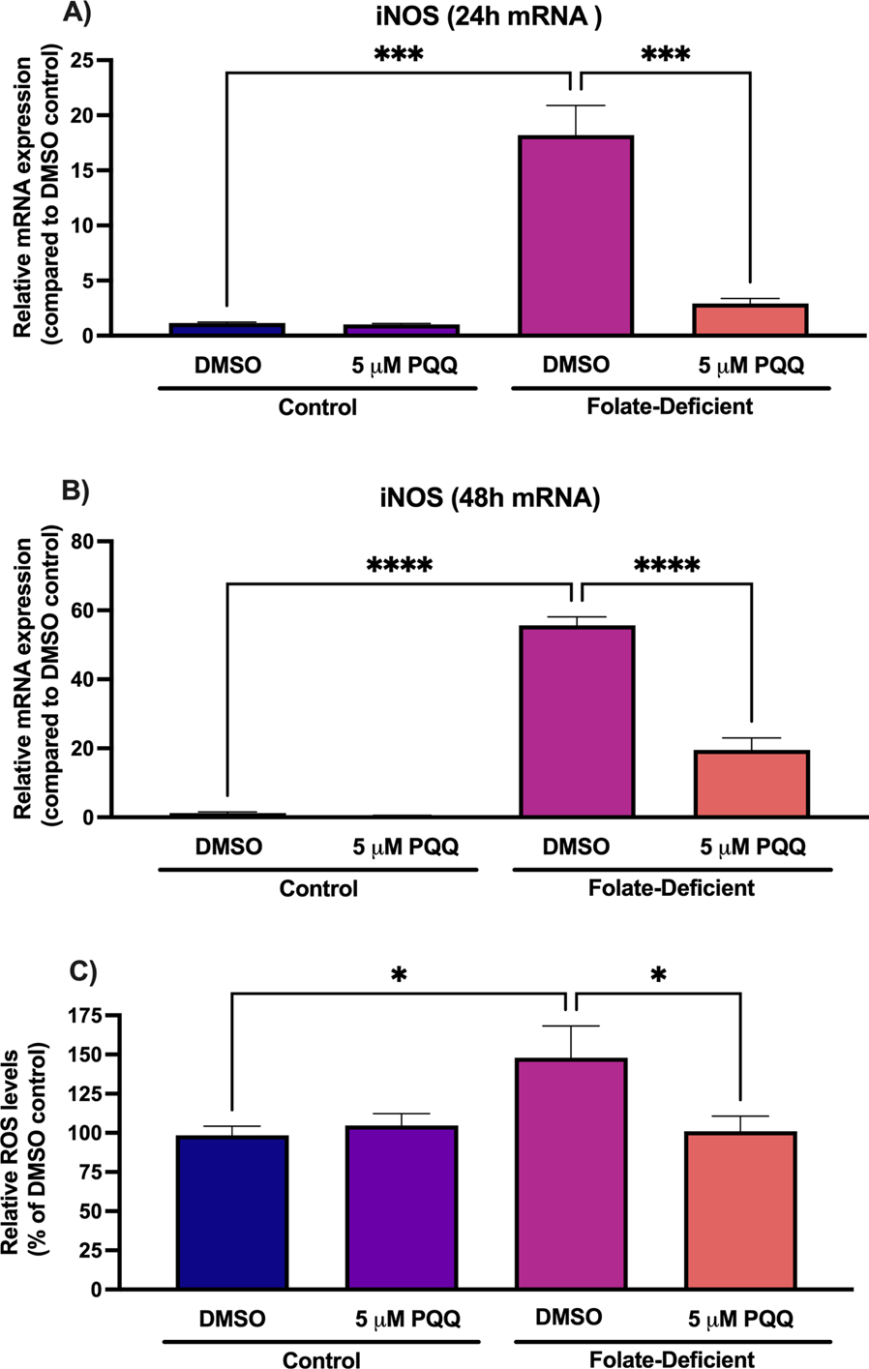
Effect of folate deficiency on oxidative stress markers in primary cultures of mouse mixed glial cells. **A**, **B** iNOS gene expression was significantly increased in FD cells compared to control, with a significant reduction in gene expression observed in FD cells following 24- and 48-h PQQ treatment (5 µm) compared to vehicle. Results are presented as mean relative mRNA expression compared to the DMSO vehicle control ± SEM normalized to the housekeeping gene cyclophilin B from N = 5 independent mixed glial preparations. **C** Relative ROS levels (oxidation of DCFH) were significantly increased in FD cells compared to vehicle control, with a significant reduction observed following 24-h PQQ treatment (5 µm). Results are presented as a percentage of relative fluorescence units normalized to the vehicle control ± SEM from N = 4 independent mixed glial preparations. One-way ANOVA with Bonferroni’s post-hoc test. Asterisks represent significant differences (*P < 0.05, **P < 0.01, ***P < 0.001, ****P < 0.0001)

To examine the role of folate deficiency in inducing inflammatory responses in vitro, changes in the gene expression of several proinflammatory cytokines and chemokines was assessed in FD mixed glial cells. Additionally, the effects of PQQ treatment in reversing these effects was examined. Through qPCR analysis, changes in gene expression of the proinflammatory cytokines and chemokines IL-1β, IL-6, CXCL10 and CCL3 was analyzed. In the FD condition, gene expression of all inflammatory markers assessed was significantly increased at 24 h (IL-1β: ~ fourfold, IL-6: ~ 14-fold, CXCL10: ~ tenfold, CCL3: ~ 15-fold) and at 48 h (IL-1β: ~ sixfold, IL-6: ~ 13-fold, CXCL10: ~ 15-fold, CCL3: ~ 18-fold) (Fig. 2). PQQ treatment (24- and 48-h) effectively mitigated these effects compared to vehicle control but was unable to restore expression levels to those seen in control cells (Fig. 2). Furthermore, the effects of folate deficiency on inducing oxidative stress responses were assessed in mixed glial cells. Gene expression of the oxidative stress marker inducible nitric oxide synthase (iNOS) was significantly and robustly increased in FD cells (24 h: ~ 18 fold, 48 h: ~ 55-fold) compared to control, with PQQ treatment (24 and 48 h) significantly reducing these effects (Fig. 3A, B). When assessing changes in ROS production, cells grown in FD conditions displayed modest but significant increases in cellular ROS levels compared to control, with increases of ~ 50% observed (Fig. 3C). Treatment with PQQ was able to mitigate these effects and restore these levels to baseline (Fig. 3C).

### Effects of folate-deficiency and PQQ treatment on mitochondrial function in mixed glial cells

**Fig. 4:**
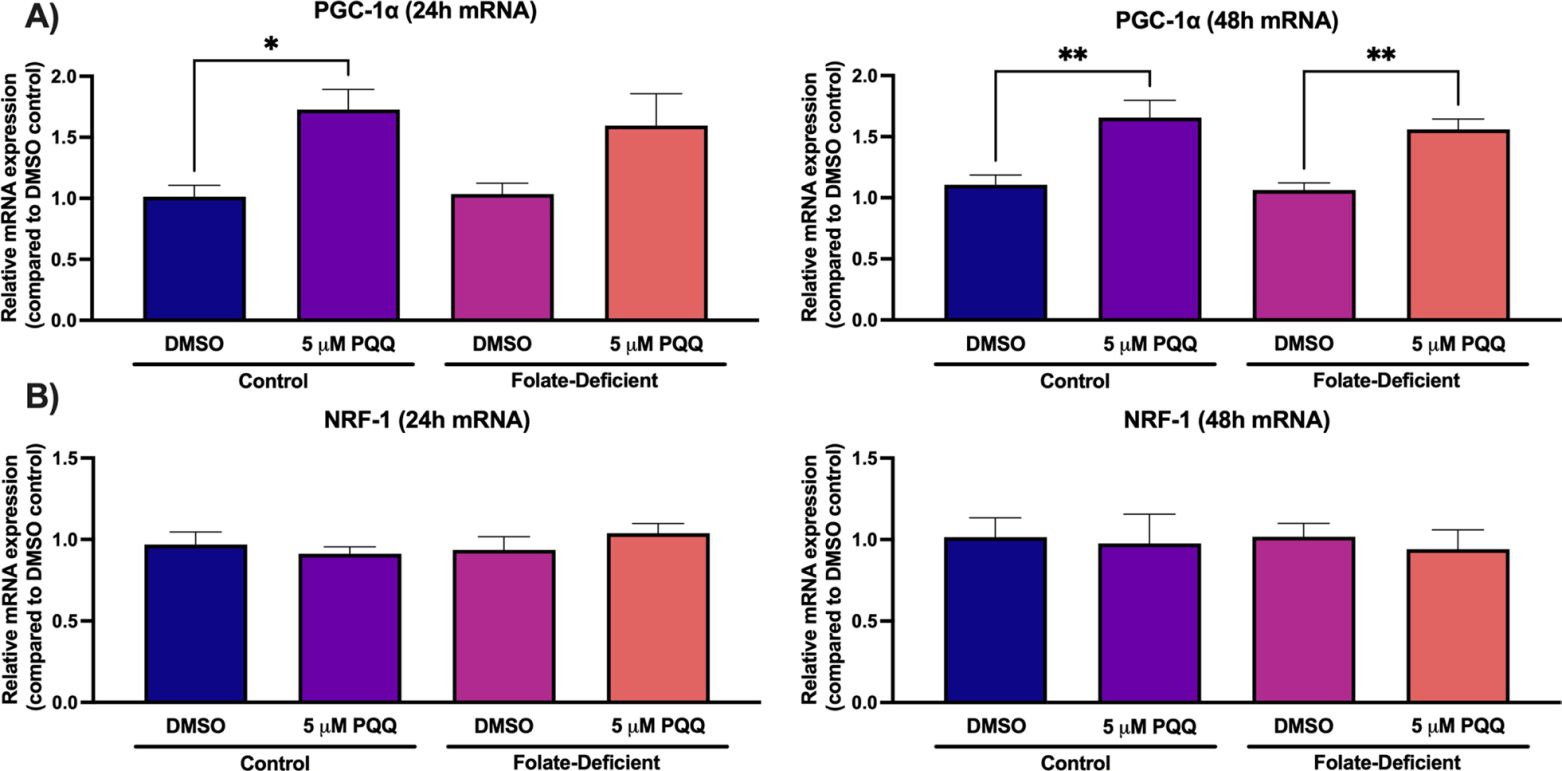
Effect of folate deficiency and PQQ treatment on PGC-1α and NRF-1 gene expression in primary cultures of mouse mixed glial cells. **A** PGC-1α gene expression was significantly increased in control and FD cells following 48-h PQQ treatment (5 µM). **B** NRF-1 gene expression was unchanged following 24- and 48-h PQQ treatment (5 µM). Results are presented as mean relative mRNA expression compared to the DMSO vehicle control ± SEM normalized to the housekeeping gene cyclophilin B from N = 5 independent mixed glial preparations. One-way ANOVA with Bonferroni’s post-hoc test. Asterisks represent significant differences (*P < 0.05, **P < 0.01)

**Fig. 5:**
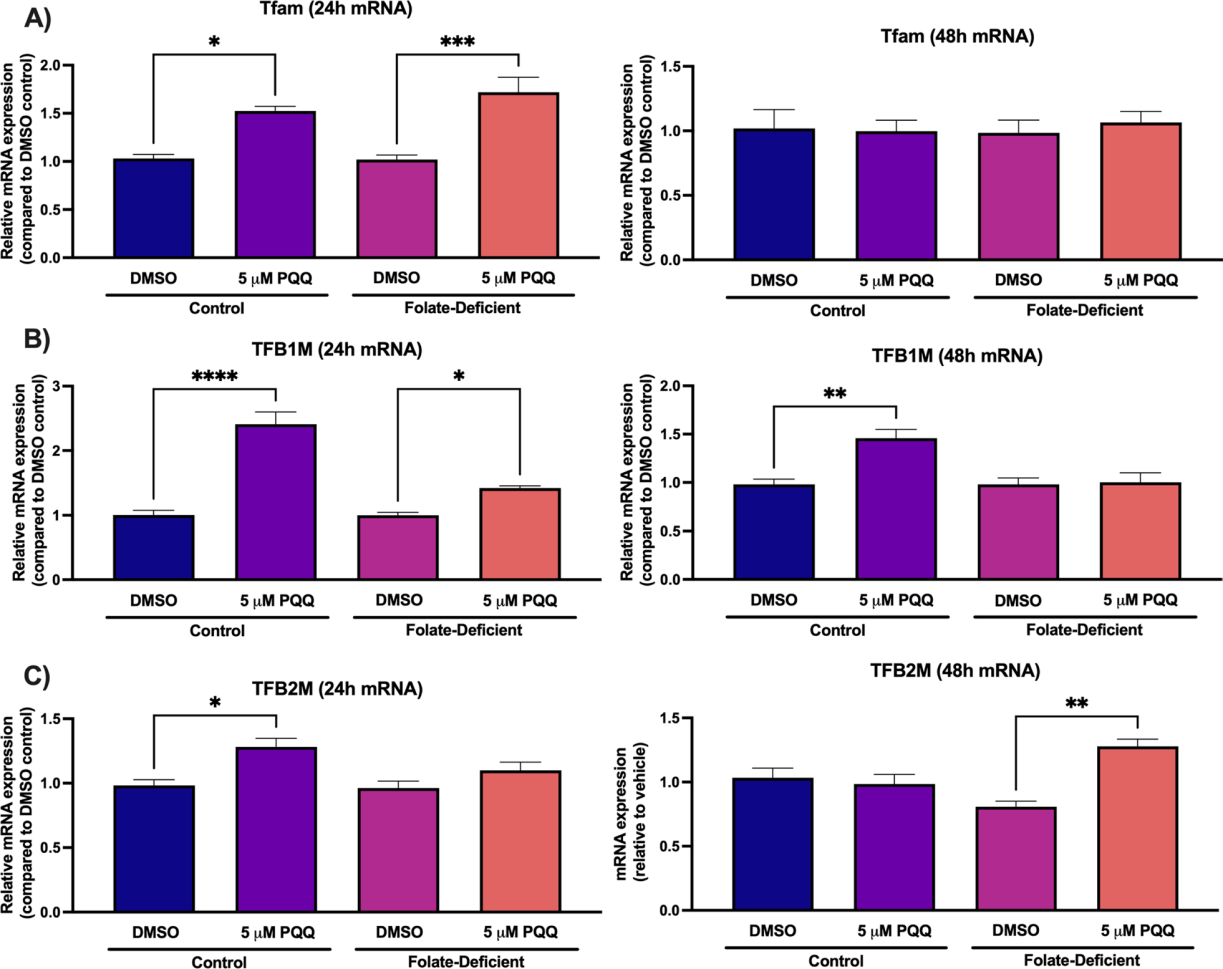
Effect of folate deficiency and PQQ treatment on gene expression of NRF-1 target genes in primary cultures of mouse mixed glial cells. **A** Tfam gene expression was significantly increased in FD cells following 24-h PQQ treatment (5 µM). **B** TFB1M gene expression was significantly increased in control and FD cells following 24-h PQQ treatment (5 µM), and in control cells following 48-h PQQ treatment (5 µM). **C** TFB2M gene expression was significantly increased in FD cells following 48-h PQQ treatment (5 µM). Results are presented as mean relative mRNA expression compared to the DMSO vehicle control ± SEM normalized to the housekeeping gene cyclophilin B from N = 5 independent mixed glial preparations. One-way ANOVA with Bonferroni’s post-hoc test. Asterisks represent significant differences (*P < 0.05, **P < 0.01)

**Fig. 6:**
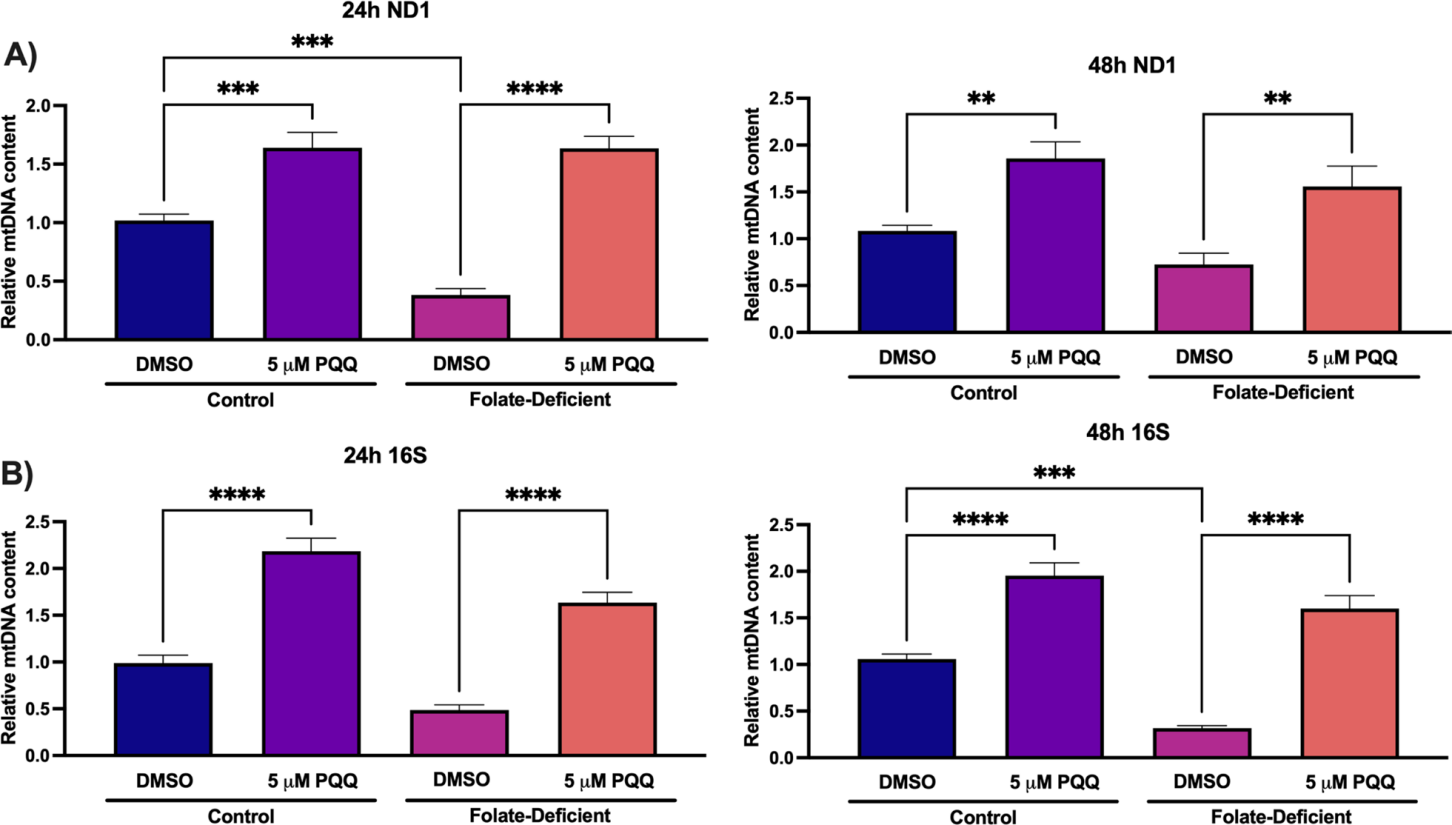
Effect of folate deficiency on relative mtDNA content in primary cultures of mouse mixed glial cells. **A** Relative ND1 expression was significantly decreased in FD cells (at 24-h), with 24 and 48-h PQQ treatment resulting in significant increases in mtDNA content in control and FD cells.** B** Relative 16S expression was significantly decreased (at 48-h) with 24 and 48-h PQQ treatment resulting in significant increases in mtDNA content in control and FD cells. Results are presented as mean ± SEM from N = 5 independent mixed glial preparations. One-way ANOVA with Bonferroni’s post-hoc test. Asterisks represent significant differences (*P < 0.05, **P < 0.01, ***P < 0.001, ****P < 0.0001)

The role of folate deficiency in inducing changes in mitochondrial function was examined by measuring changes in gene expression of the PGC-1α/NRF-1 signaling cascade, as well as changes in relative mtDNA content. In all PGC-1α/NRF-1 signaling genes assessed, no significant changes in expression were observed between control and FD cells (Figs. 4, 5). However, PQQ treatment led to significant increases in PGC-1α gene expression in control cells at 24 h by ~ 70% and 48 h by ~ 60%, with increased expression observed in FD cells only at 48 h by ~ 50% (Fig. 4A). Conversely, NRF-1 transcript levels remained unchanged following PQQ treatment (Fig. 4B). These results were anticipated as PQQ treatment did not affect NRF-1 expression levels in our previous in vitro studies in hCMEC/D3 cells [21, 22]. To further investigate activation of the PGC-1α/NRF-1 signaling pathway, effects of PQQ on gene expression of known NRF-1 downstream targets mitochondrial transcription factor A (Tfam) and B (TFB1M, TFB2M) were also measured. In control and FD cells, significant increases in Tfam gene expression were observed following the 24-h PQQ treatment (control: ~ 50%, FD: ~ 70%) (Fig. 5A). TFB1M gene expression was increased in control and FD cells following the 24-h PQQ treatment (control: ~ 2.5-fold, FD: ~ 40%), however a significant increase was only observed in control cells following the 48-h treatment by ~ 45% (Fig. 5B). TFB2M gene expression was significantly increased in control cells following the 24-h PQQ treatment by ~ 25%, with expression only significantly increasing in FD cells following the 48-h treatment by ~ 30% (Fig. 5C). Lastly, the effects of folate deficiency and PQQ treatment was analyzed on changes in relative mtDNA content by measuring changes in gene expression of two known mtDNA encoded genes, ND1 and 16S. Exposure to folate deficiency led to significant reductions in relative ND1 expression at 24 h (~ 60%), with a reduction of 16S expression observed in FD cells at 48 h (~ 75%) (Fig. 6A, B). However, PQQ led to significant increases in ND1 expression in both control and FD cells at 24 h (control: ~ 60%, FD: ~ 60%) and at 48 h (control: ~ 60%, FD: ~ 50%) (Fig. 6A). In addition, PQQ treatment led to significant increases in 16S expression in both control and FD cells at 24 h (control: ~ twofold, FD: ~ 60%) and 48 h (control: ~ 80%, FD: ~ 60%) (Fig. 6B).

### Effects of folate deficiency and PQQ treatment on changes in folate transporter regulation and inflammatory processes in wildtype mice

**Fig. 7:**
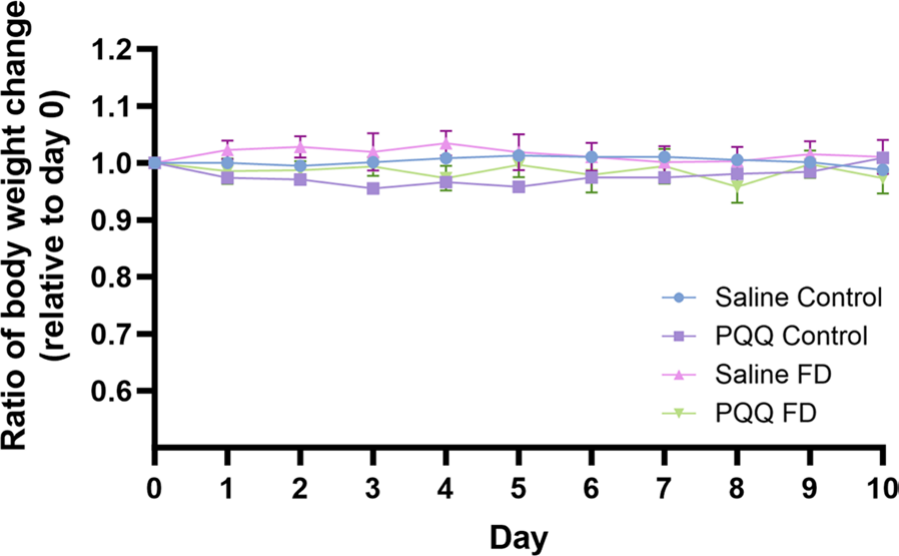
Effect of in vivo PQQ treatment in wildtype mice. Changes in body weight (relative to day 0) was measured over the course of the 10-day PQQ (20 mg/kg/day i.p) or saline vehicle treatment period in mice assigned to control (2 mg/kg folate) and FD (0 mg/kg folate) diets. No significant changes in weight were observed between control mice and FD mice, with no changes in weight observed following PQQ treatment compared to saline vehicle. Results are presented as mean change in body weight ± SEM from N = 5–6 mice

**Fig. 8:**
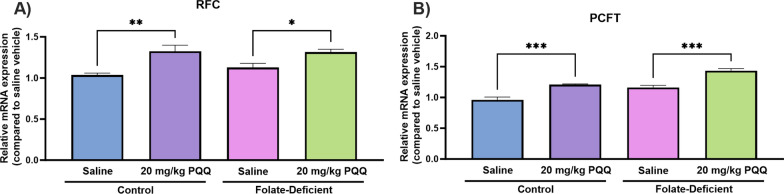
Effect of folate deficiency and PQQ treatment on the gene expression of the folate transporters RFC (*Slc19a1*) and PCFT (*Slc46a1*) in whole brains of wildtype mice assigned to control (2 mg/kg folate) or FD (0 mg/kg folate) diets. **A** RFC gene expression was significantly increased in control and FD mouse brains following the 10-day PQQ treatment (20 mg/kg/day) compared to saline vehicle.** B** PCFT gene expression was significantly increased in control and FD mouse brains following the 10-day PQQ treatment (20 mg/kg/day) compared to saline vehicle. Results are presented as mean relative mRNA expression compared to the saline vehicle ± SEM normalized to the housekeeping gene cyclophilin B from N = 5–6 mouse brains. One-way ANOVA with Bonferroni’s post-hoc test. Asterisks represent significant differences (*P < 0.05, **P < 0.01, ***P < 0.001)

**Fig. 9:**
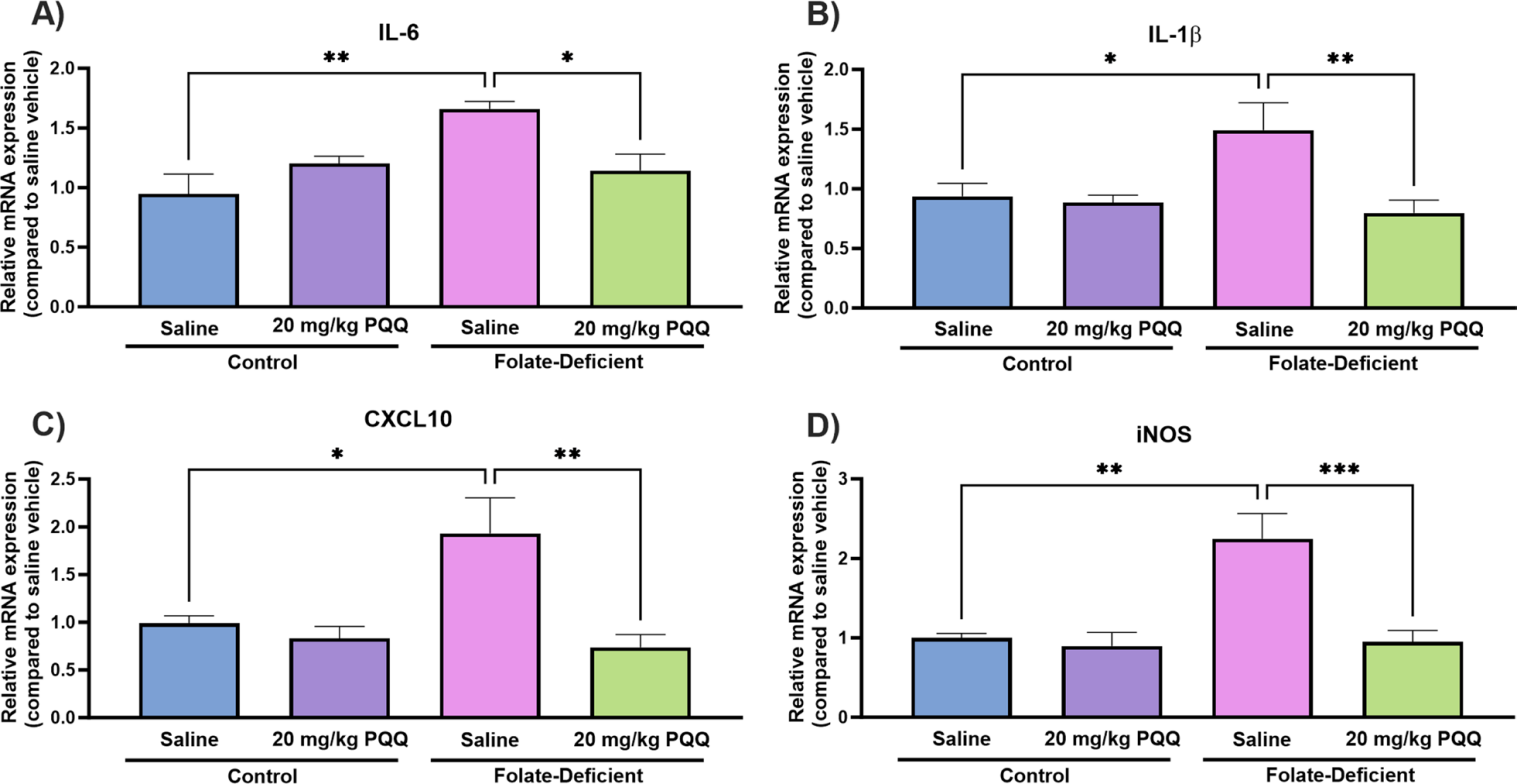
Effect of folate deficiency and PQQ treatment on the gene expression of inflammatory and oxidative stress markers in whole brains of wildtype mice assigned to control (2 mg/kg folate) or FD (0 mg/kg folate) diets.** A**–**D** IL-6, IL-1β, CXCL10, and iNOS gene expression was significantly increased in brains of saline-treated FD mice, with the 10-day PQQ treatment resulting in decreased marker expression in FD mouse brains compared to saline control. Results are presented as mean relative mRNA expression compared to the saline vehicle control ± SEM normalized to housekeeping gene cyclophilin B from N = 5–6 mouse brains. One-way ANOVA with Bonferroni’s post-hoc test. Asterisks represent significant differences (*P < 0.05, **P < 0.01, ***P < 0.001)

**Fig. 10:**
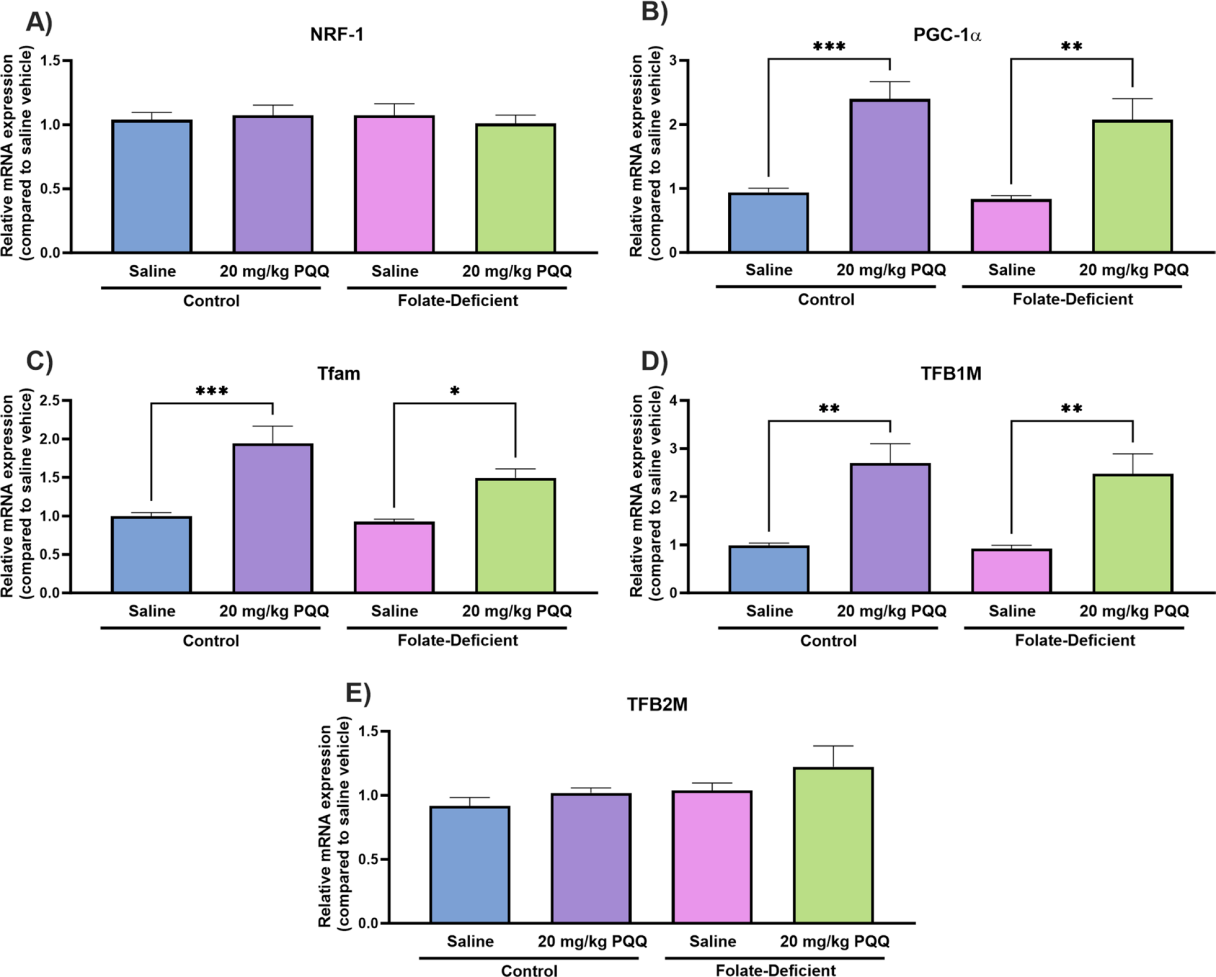
Effect of folate deficiency and PQQ treatment on the gene expression of PGC-1α/NRF-1 signaling genes in whole brains of wildtype mice assigned to control (2 mg/kg folate) or FD (0 mg/kg folate) diets. **A** NRF-1 gene expression remained unchanged in control and FD mouse brains following PQQ treatment compared to saline control. **B**-**D** PGC-1α, Tfam, and TFB1M gene expression was significantly increased in control and FD mouse brains following PQQ treatment compared to saline vehicle. **E** TFB2M gene expression was unchanged in control and FD mouse brains following PQQ treatment compared to saline control. Results are presented as mean relative mRNA expression compared to the saline vehicle ± SEM normalized to the housekeeping gene cyclophilin B from N = 5–6 mouse brains. One-way ANOVA with Bonferroni’s post-hoc test. Asterisks represent significant differences (*P < 0.05, **P < 0.01, ***P < 0.001)

To determine whether folate deficiency may induce similar physiological impairments in vivo, male wildtype mice (C57BL6/N) were placed on a 5-week FD (or control) diet, followed by a 10-day PQQ (20 mg/kg/day i.p) or saline treatment regimen. Mouse brains were later isolated 24 h following the last injection, and tissue was processed to analyze changes in gene expression of the folate transport systems, inflammatory and oxidative stress markers, as well as changes in PGC-1α/NRF-1 signaling genes. Previous studies in our laboratory demonstrated the effects of PQQ in vivo*,* in wildtype mice, with a 10-day PQQ treatment regimen (10 mg/kg/day, i.p.) resulting in modest but significant increases in RFC gene expression in isolated brain capillaries [21, 22]. Our current studies employed a higher dose PQQ dose to elicit more robust changes in RFC expression in whole brains of wildtype mice, along with inducing anti-inflammatory and antioxidant effects. As our studies utilized a PQQ dose of 20 mg/kg/day, we measured changes in body weight over the 10-day treatment period to determine whether this dose may result in any unintended toxicities. Over this treatment period, no significant changes in body weight were observed between PQQ and saline-treated mice (Fig. 7). In addition, there were no significant changes in body weight between mice placed on FD diets in comparison to mice on control diets (Fig. 7). In PQQ-treated mice, significant elevations in RFC (control: ~ 30%, FD: ~ 30%) and PCFT (control: ~ 15%, FD: ~ 25%) gene expression was observed in both control and FD groups (Fig. 8A, B). Interestingly, no differences in PCFT gene expression were observed in FD mouse brains compared to control, which contrasts with our data in mixed glial cells (Fig. 8B). When assessing inflammatory and oxidative stress markers, significant increases in gene expression of IL-6 (~ 60%), IL-1β (~ 50%), CXCL10 (~ 90%), and iNOS (~ twofold) was observed in the brains of saline-treated FD mice (Fig. 9). However, PQQ treatment was able to mitigate these increases, effectively restoring them to baseline levels (Fig. 9). To confirm activation of the PGC-1α/NRF-1 signaling cascade following PQQ treatment, gene expression of NRF-1, PGC-1α, and downstream NRF-1 targets (Tfam, TFB1M, TFB2M) was examined. In both control and FD mouse brains, a significant increase in PGC-1α expression was observed (control: ~ 50%, FD: ~ 35%), with NRF-1 levels remaining unchanged (Fig. 10A, B). However, a significant increase in Tfam (control: ~ twofold, FD: ~ 50%) and TFB1M (control: ~ 2.5-fold, FD: ~ 2.3-fold) levels were observed in both control and FD mouse brains following PQQ treatment (Fig. 10C, D). However, expression levels of TB2M remained unchanged following PQQ treatment in both control and FD mouse brains.

## Discussion

Folates play a significant role in many critical biosynthetic and homeostatic processes in the CNS and systemic tissues [1]. As adequate folate concentrations are required for normal cellular growth and development, folate deficiency has been identified as a risk factor in several disorders including neural tube defects, cardiovascular disorders, megaloblastic anemia, and CFD [52]. While disorders of CFD (both primary and secondary) are characterized by suboptimal CSF folate levels, the presence of brain inflammation, oxidative stress, and mitochondrial dysfunction has become increasingly recognized [18, 39, 40]. However, whether folate deficiency may play a significant role in inducing these changes has not been thoroughly investigated. Several studies have described the benefits of PQQ and its potential use in treatment strategies for neurological disorders [25, 27, 28, 33]. Our group has reported the significant upregulation of RFC by PQQ at the BBB, hence, this compound could augment folate delivery to the brain in disorders associated with impaired folate transport to the CNS, while in parallel providing additional protective effects to alleviate the severe neurological impairments often seen in disorders of CFD [21, 22]. The purpose of this study was to further investigate the role of brain folate deficiency in inducing changes in inflammation, oxidative stress, and mitochondrial dysfunction that may further contribute to the pathophysiological features of CFD disorders and examine the potential role of PQQ in eliciting protective effects.

We first investigated whether folate deficiency may result in changes in expression of the folate transport systems, and the role of PQQ in upregulating RFC and PCFT expression in mixed glial cells and the brains of wildtype mice. Compared to control, gene expression of PCFT was significantly decreased in FD cells, with no changes observed in RFC expression (Fig. 1). Interestingly, this PCFT downregulation was not observed in the brains of FD mice (Fig. 8). While the effects of folate deficiency on the expression of the folate transport systems have been previously demonstrated, our current study provides novel evidence of changes in folate transporter expression in astrocytes and microglia in response to folate deficiency. In the study by Thakur et al. an intestinal PCFT upregulation was observed in FD human Caco-2 cells [53]. In contrast, Yang et al. reported increased RFC gene expression and decreased PCFT gene expression in FD mouse hippocampal neuronal cells (HT-22), which suggests that this decreased PCFT expression in response to folate deficiency may be species-specific and restricted to the CNS [54]. In the present study, treatment with PQQ led to significant increases in both RFC and PCFT gene expression in mouse mixed glial cells and the brains of wildtype mice, which agrees with our previously published data at the BBB (Fig. 1, 8) [21, 22]. It is important to note that in the context of CFD, PCFT may not serve as a relevant transporter as it displays optimal activity at acidic pH, and therefore may not play a significant role in increasing cerebral folate uptake outside of the choroid plexus epithelium [9]. To our knowledge, these results represent the first report of PGC-1α/NRF-1-mediated upregulation of RFC in mixed glial cells, which further complement our previous studies documenting similar effects at the human BBB [21, 22].

In several neurological disorders including disorders associated with CFD, the presence of inflammation and oxidative stress in the brain can contribute to impaired neurological function, ultimately resulting in cognitive deficits [39, 40, 55]. In these studies, we measured changes in gene expression of several proinflammatory cytokines, chemokines and oxidative stress markers in mixed glial cells and brains of wildtype mice, as well as examining changes in ROS levels in vitro in response to folate deficiency and PQQ treatment. We observed robust increases in gene expression of several inflammatory and oxidative stress markers both in vitro, in FD cells, and in vivo, in brains of mice exposed to FD diets. PQQ treatment in vivo was able to attenuate these increased expression levels, with PQQ treatment in vivo effectively decreasing expression levels of those seen in control mice (Fig. 2, 9). In addition, a modest elevation in cellular ROS levels was observed in mixed glial cells under conditions of folate deficiency. PQQ treatment elicited antioxidant effects, leading to a reduction in ROS levels observed in vitro (Fig. 3).

The direct effects of folate deficiency in inducing inflammatory and oxidative stress are not well-understood, with studies in the literature primarily focused on examining its role in enhancing these responses in the presence of infection, disease, or cell injury. In the study by Cheng et al. folate deficiency enhanced the immune response of oxygen–glucose deprivation-treated BV2 microglial cells resulting in increased protein expression of TNFα, IL-1β, and IL-6 [37]. Similar results were observed in the mouse monocyte cell line RAW264.7, with FD cells demonstrating enhanced expression of several pro-inflammatory mediators at the gene and protein level following lipopolysaccharide (LPS) treatment [34]. In our studies, increases in inflammatory responses were observed in the absence of additional cellular stressors, suggesting that folate deficiency itself may be sufficient to induce neuroinflammation in disorders of CFD.

The induction of oxidative stress in response to folate deficiency has also been previously reported through increases in ROS levels, as well as reduced expression and activity of antioxidant enzymes (i.e., catalase, sodium dismutase) [35]. In an in vitro FD model, HepG2 cells grown in media lacking folic acid displayed significant increases in thiobarbituric acid reactive substances (TBARS) levels (index of lipid peroxidation), as well as increased hydrogen peroxide levels [35]. These effects are proposed to be the result of activation of the NF-κB pathway, which has been recognized for its role in inducing the gene expression of proinflammatory and pro-oxidant mediators [37, 56].

The anti-inflammatory and antioxidant effects of PQQ have been extensively investigated, with several in vitro and in vivo studies demonstrating a reduction in the expression of proinflammatory cytokines and chemokines in response to PQQ treatment, along with decreases in markers of oxidative stress ([30, 57]). Our studies however provide the first evidence of PQQ eliciting protective effects in a model of brain folate deficiency. In LPS-treated microglia cells, PQQ treatment has led to a suppression of several pro-inflammatory and oxidative stress mediators (i.e., iNOS, IL-1β, and IL-6), with PQQ treatment eliciting similar anti-inflammatory effects in the brains of LPS-treated mice [30]. In a mouse D-galactose model, PQQ elicited antioxidant effects by reducing MDA and ROS levels, with increases in SOD2 gene expression also observed [57]. As seen with FD, PQQ may elicit its anti-inflammatory and antioxidant effects through modulation of the NF-κB and Nrf2/Keap1 pathway, respectively [58]. Furthermore, PQQ treatment may result in significant improvements in cognitive function in neurological disorders, with PQQ supplementation preventing cognitive deficits in an MK-801-induced schizophrenia mouse model [59]. As many disorders of CFD are also characterized by a marked reduction in neurological function, PQQ may serve as a promising therapeutic compound in alleviating any associated cognitive deficits.

It is proposed that mitochondrial dysfunction may play a significant role in many of these neurological disorders associated with CFD, resulting in the inability to meet the metabolic demands of the CNS [40]. In our studies in mouse mixed glial cells and brains of wildtype mice, PQQ treatment led to significant increases in PGC-1α gene expression, but not NRF-1 expression, which was previously observed in our studies in hCMEC/D3 cells (Fig. 4, 10) [21, 22]. Similar results were observed in our studies examining the effects of VDR upregulation on RFC functional expression, as treatment with the VDR ligand calcitriol did not induce VDR expression [19]. However, expression of NRF-1 downstream target genes was significantly upregulated following PQQ treatment, which suggests that PQQ treatment resulted in NRF-1 activation (Fig. 5, 10). We also determined whether there were any differences in expression of PGC-1α/NRF-1 signaling genes between control and FD conditions both in vitro and in vivo, as Chang et al. had observed significant upregulation in genes implicated in mitochondrial biogenesis (i.e., PGC-1α, NRF-1, Tfam) in rats exposed to folate deprivation [38]. Interestingly, there were no significant differences in gene expression of any of the PGC-1α/NRF-1 signaling genes investigated in mixed glial cells and brains of wildtype mice (Figs. 5, 10). As folate deficiency is also associated with mtDNA instability, changes in mtDNA content were assessed in control and FD cells. Several mtDNA encoded mitochondrial enzymes play a critical role in the electron transport chain (ETC), therefore mtDNA degradation can result in impaired mitochondrial function [60]. As the ETC serves as a significant ROS source, disruptions in ATP production can result in increased oxidative stress [61]. In our studies, changes in mtDNA content were assessed in control and FD cells by analyzing changes in expression of two mtDNA-encoded genes, 16S and ND1 [51]. In FD cells, significant decreases of relative mtDNA content were observed, which has been previously demonstrated in FD rodents (Fig. 4) [36, 38]. However, treatment with PQQ significantly increased mtDNA content in both control and FD cells, indicative of increased mitochondrial biogenesis (Fig. 4). As PGC-1α functions as the master regulator of mitochondrial biogenesis, the effects of PQQ in increasing mtDNA content has been well-established [25]. As mitochondrial biogenesis is a tightly regulated process that increases total mitochondria in response to metabolic demand, it is anticipated that upregulating mitochondrial biogenesis through PGC-1α/NRF-1 activation by PQQ may provide benefits in increasing overall CNS function [62].

We are cognizant of the limitations of the present study, as our in vitro and in vivo findings primarily document changes in gene expression of the markers assessed. As the in vitro work was conducted in primary cultures of mixed glial cells, obtaining sufficient cell yield remains a challenge to perform additional biochemical assays. With respect to changes in protein expression of the folate transport systems, our previous studies at the human BBB have documented that PGC-1α/NRF-1 activation results in significant increases in protein expression as well as function, therefore we anticipate similar results in brain parenchymal cells [21, 22]. Despite these limitations, the results from our models are consistent in their demonstration of the physiological changes induced by folate deficiency, as well as the neuroprotective effects of PQQ treatment.

## Conclusions

Taken together, our studies have revealed for the first time the direct effects of folate deficiency on inducing inflammatory and oxidative stress responses, as well as mitochondrial dysfunction, which may contribute to the neurological impairments commonly observed in disorders associated with CFD. Our in vitro and in vivo studies demonstrate the direct effects of PQQ attenuating the effects of folate deficiency in the absence of folate re-supplementation. While we have previously shown the effects of PQQ in inducing RFC function at the BBB, our current studies demonstrate novel upregulation of RFC in brain parenchymal cells, which may assist in enhanced folate uptake into glial cells, and thus mitigating the effects of brain folate deficiency. Our data uncovers novel effects of PQQ treatment in FD models, effectively reversing the changes in inflammation, oxidative stress, and mitochondrial dysfunction caused by folate deficiency both in vitro and in vivo, suggesting that it may additionally provide neuroprotective effects while in parallel increasing the transport of folates in brain microvessel endothelial cells, as well as in glial cells. As current treatments for CFD are limited (i.e., folinic acid treatment), PQQ may be quite beneficial to be used in conjunction with standard folate supplementation therapies, thereby enhancing the transport of folinic acid, while providing additional protective effects. Future studies will be conducted to examine whether these FD-induced changes can result in behavioural impairments like those seen in disorders of CFD, and whether PQQ treatment may alleviate any behavioural deficits that arise because of brain folate deficiency.

### Supplementary Information

**Additional file 1: Figure S1.** Effect of PQQ treatment on control and FD primary cultures of mouse mixed glial cells. An MTT assay was used to confirm the effects of various PQQ doses (5-50 μM for 48 h) on cell viability in mixed glial cells. **A** Exposure to 50 μM PQQ significantly reduces cell viability compared to untreated cells in the control condition. **B** Exposure to 50 μM PQQ significantly reduces cell viability compared to untreated cells in the FD condition. Results are presented as mean ± S.E.M. for N=3 independent experiments. One-way ANOVA with Bonferroni’s post-hoc test. Asterisks represent significant differences (***P < 0.0001, ****P 0.00001).

## Data Availability

The datasets used and/or analysed during the current study are available from the corresponding author on reasonable request.

## Abbreviations

Calcitriol: 1,25 Dihydroxyvitamin D3
16S: 16S ribosomal RNA
AB: Arachnoid barrier
ASD: Autism spectrum disorder
BBB: Blood–brain barrier
BCSFB: Blood-cerebrospinal fluid barrier
CAT: Catalase
CFD: Cerebral folate deficiency
CNS: Central nervous system
CSF: Cerebrospinal fluid
DCFH: 2′,7′-Dichlorofluorescein
DMEM: Dulbecco’s Modified Eagle Medium
DNA: Deoxyribonucleic acid
FD: Folate-deficient
FRAAs: Folate receptor autoantibodies
FRα: Folate receptor alpha
GFAP: Glial fibrillary acidic protein
GPx: Glutathione peroxidase
hCMEC/d3: Human brain microvessel endothelial cells
HFM: Hereditary folate malabsorption
HK2: Hexokinase 2
IBA1: Ionized calcium binding adaptor molecule 1
iNOS: Inducible nitric oxide synthase
Keap1: Kelch-like ECH-associated protein 1
KSS: Kearne Sayre Syndrome
MDA: Malondialdehyde
mtDNA: Mitochondrial DNA
MTT: Teratozium salt
ND1: NADH-ubiquinone oxidoreductase chain 1
NF-kB: Nuclear factor kappa beta
NRF-1: Nuclear respiratory factor 1
Nrf2: Nuclear factor erythroid 2-related factor 2
PCFT: Proton-coupled folate transporter
PGC-1α: Peroxisome proliferator-activated receptor-γ coactivator-1α
PQQ: Pyrroloquinoline quinone
RFC: Reduced folate carrier
RNA: Ribonucleic acid
ROS: Reactive oxygen species
SOD: Superoxide dismutase
TBARS: Thiobarbituric acid reactive substances
Tfam: Mitochondrial transcription factor A
TFB1M/2 M: Mitochondrial transcription factor B
VDR: Vitamin D Receptor
