# Supplementary material for: Protective effects of pyrroloquinoline quinone in brain folate deficiency
Source: Fluids Barriers CNS. 2023 Nov 20;20:84. doi: 10.1186/s12987-023-00488-3 (PMC10659058; doi:10.1186/s12987-023-00488-3)
Supplement: Supplementary file 1 — Additional file 1: Figure S1. Effect of PQQ treatment on control and FD primary cultures of mouse mixed glial cells. An MTT assay was used to confirm the effects of various PQQ doses (5-50 μM for 48 h) on cell viability in mixed glial cells. A Exposure to 50 μM PQQ significantly reduces cell viability compared to untreated cells in the control condition. B Exposure to 50 μM PQQ significantly reduces cell viability compared to untreated cells in the FD condition. Results are presented as mean ± S.E.M. for N=3 independent experiments. One-way ANOVA with Bonferroni’s post-hoc test. Asterisks represent significant differences (***P < 0.0001, ****P 0.00001). [file 12987_2023_488_MOESM1_ESM.docx]

**Supplemental Information**

**Supplemental Figure:**

**Figure S1:** Effect of PQQ treatment on control and FD primary cultures of mouse mixed glial cells. An MTT assay was used to confirm the effects of various PQQ doses (5-50 μM for 48h) on cell viability in mixed glial cells. A) Exposure to 50 μM PQQ significantly reduces cell viability compared to untreated cells in the control condition. B) Exposure to 50 μM PQQ significantly reduces cell viability compared to untreated cells in the FD condition. Results are presented as mean ± S.E.M. for *n* = 3 independent experiments. One-way ANOVA with Bonferroni’s post-hoc test. Asterisks represent significant differences (***P<0.0001, ****P 0.00001).
